# Goblet Cell Adenocarcinoma in the Stomach: A Case Report

**DOI:** 10.7759/cureus.58592

**Published:** 2024-04-19

**Authors:** Yasunori Enomoto, Yoshifumi Arai, Shiori Meguro, Hideya Kawasaki, Isao Kosugi, Toshihide Iwashita

**Affiliations:** 1 Department of Regenerative and Infectious Pathology, Hamamatsu University School of Medicine, Hamamatsu, JPN; 2 Pathology, Toyohashi Municipal Hospital, Toyohashi, JPN; 3 Preeminent Medical Photonics Education and Research Center Institute for NanoSuit Research, Hamamatsu University School of Medicine, Hamamatsu, JPN

**Keywords:** malignancy surgery, neuroendocrine neoplasm, goblet cell adenocarcinoma, gastrointestinal pathology, rare gastric tumor

## Abstract

Goblet cell adenocarcinoma (GCA) is known as an amphicrine tumor often seen in the appendix. Here, we report a rare case of GCA in the stomach. An 80-year-old man underwent gastroscopy due to epigastric pain and was diagnosed with gastric cancer. He received total gastrectomy and histology showed a mixture of a moderately-differentiated tubular adenocarcinoma, a mucinous adenocarcinoma, and a tumor composed of goblet-like mucinous cells with neuroendocrine differentiation. The tumor volume ratio was about 4:1:5, respectively, and a final diagnosis of GCA was made. The metastasis of the regional lymph node was occupied by only the component of goblet-like cells. GCA should be recognized as a rare histologic subtype of gastric cancer.

## Introduction

Goblet cell adenocarcinoma (GCA) is a rare malignancy often detected in the appendix, which is defined as an amphicrine tumor composed of goblet-like mucinous cells, as well as variable numbers of endocrine cells and Paneth-like cells [1]. Historically, it has been called by various names such as goblet cell carcinoid, adenocarcinoid, mucinous carcinoid, and mucin-producing neuroendocrine tumor [1,2]. Here, we report a rare case of GCA seen in the stomach, which would be the sixth case in the English literature.

## Case presentation

**Figure 1:**
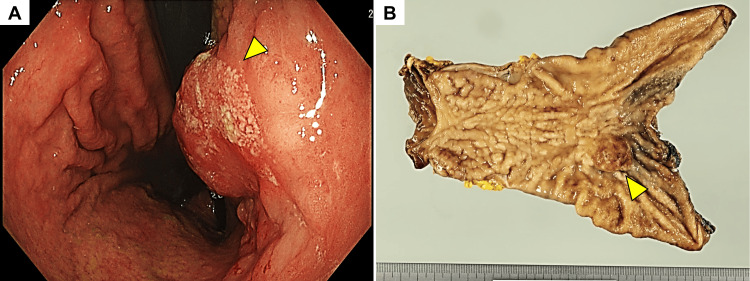
Macroscopic images of the gastric tumor A) A protruding tumor with an ulcerated surface (yellow triangle) was seen on endoscopy. B) The tumor (yellow triangle) was located at the corpus of the resected stomach.

This is the case of an 80-year-old male patient who presented with epigastric pain and tarry stool for the past month before the consultation. The patient had no relevant medical history and did not report any other general symptoms. Endoscopic examination showed a mass with a bulging and ulcerated surface located at the corpus of the stomach ​(Figure 1A). Biopsied samples suggested a moderately-differentiated tubular adenocarcinoma. Thereafter, the patient received total gastrectomy and lymph node dissection as the treatment for gastric cancer.

**Figure 2:**
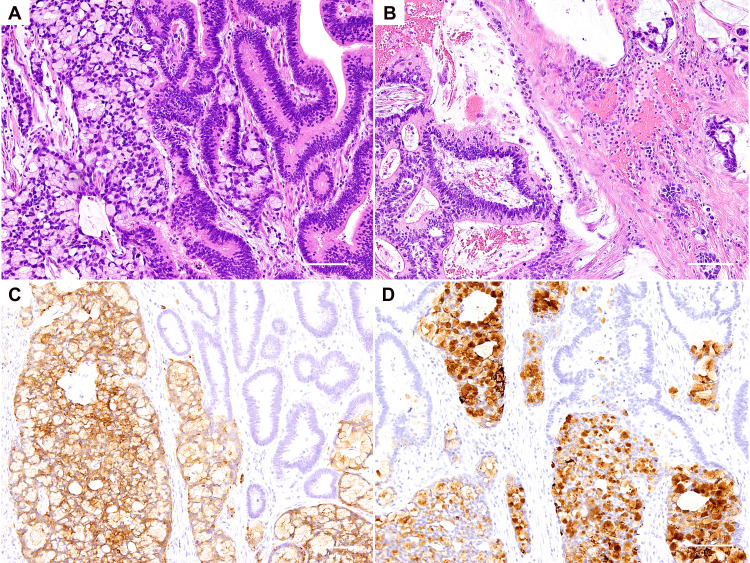
Histology and immunostaining for the gastric tumor A) A mixture of a tubular adenocarcinoma (right-sided) and a tumor of goblet-like cells (left-sided) is shown (Hematoxylin & Eosin staining, magnification: ×100, scale bar: 100 μm). B) A mixture of a mucinous adenocarcinoma (right-sided) and a tubular adenocarcinoma (left-sided) is shown (Hematoxylin & Eosin staining, magnification: ×100, scale bar: 100 μm). C) The component of goblet-like cells shows positivity for Synaptophysin (visualized using 3,3'Diaminobenzidine, magnification: ×100, scale bar: 100 μm). D) The component of goblet-like cells shows positivity for Chromogranin A (visualized using 3,3'Diaminobenzidine, magnification: ×100, scale bar: 100 μm).

**Figure 3:**
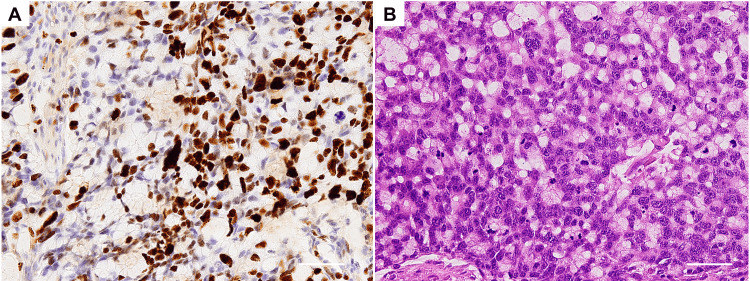
Histology and immunostaining for the component of goblet cell adenocarcinoma A) An image of Ki-67 staining in the goblet cell adenocarcinoma of the stomach is shown (visualized using 3,3'Diaminobenzidine, magnification: ×200, scale bar: 50 μm). B) Only the component of goblet-like cells is detected in the metastatic lymph node (Hematoxylin & Eosin staining, magnification: ×200, scale bar: 50 μm).

Macroscopically, the resected tumor was approximately 30×25 mm (Figure 1B). Histologically, the tumor consisted of three components: a moderately differentiated tubular adenocarcinoma, a mucinous adenocarcinoma, and a tumor composed of goblet-like mucinous cells (Figures 2A-2B). The tumor volume ratio was about 4:1:5, respectively. The component of the goblet-like cells was the most invasive and was exposed on the serosa surface. Immunohistochemical examination of the goblet-like cells showed positivity for the two neuroendocrine markers, Synaptophysin and Chromogranin A (Figures 2C-2D), which were negative on the other tumor components. Additionally, the Ki-67 proliferative index in the goblet-like cells was more than 70% (Figure 3A). Collectively, a diagnosis of GCA was established. In the non-neoplastic gastric mucosa, chronic atrophic gastritis without apparent evidence of *Helicobacter pylori* was seen. The metastasis was found in 2 of the 15 regional lymph nodes and occupied by only the goblet-like cells (Figure 3B). The tumor, node, metastasis (TNM) classification was determined as pT4aN1M0 (stage IIIB).

Six months after surgery, our follow-up was terminated because he was transferred to a chronic care hospital. At least during the period, no recurrence or metastasis was observed.

## Discussion

According to the recent WHO classification (5th) [1], GCA is classified as not a neuroendocrine neoplasm but an adenocarcinoma subtype and can include components of conventional adenocarcinomas. Additionally, the use of the term “mixed adenoneuroendocrine carcinoma (MANEC)” is no longer preferred in the context of GCA. Thus, we determined the diagnosis of the tumor in the present case as just GCA. As the tumor mainly showed tubular or clustered growth, the GCA was graded as 1 on the three-tiered system [1].

Because the majority of reports on GCA have been collected from patients with appendiceal origin, the data on GCA in the stomach is extremely scarce. To our knowledge, there have been just five cases (in four reports) describing GCA of the stomach in the English literature [3-6]. The summary of the reported cases, including ours, is shown in Table 1. Four out of the six cases were over 60 years old. The male/female ratio was 2:1. Interestingly, all the GCAs included other malignant components, such as tubular adenocarcinoma and signet-ring cell adenocarcinoma. The follow-up periods after surgery were diverse, which suggests the difficulty in discussing the prognosis, so far.

**Table 1:** Summary of reported cases with goblet cell adenocarcinoma (GCA) in the stomach

Authors	Age (y.o.)/sex	Other components	Subtypes of other components	Lymph node metastasis	Follow-up after surgery
Caruso RA, et al. [3].	60/male	Yes	Tubular adenocarcinoma; (usual) carcinoid	Not detected	No recurrence or metastasis for the following 36 months
Fujiyoshi Y, et al. [4].	41/male	Yes	Signet-ring cell adenocarcinoma	Not detected	No recurrence or metastasis for the following 12 years
Fujiyoshi Y, et al. [4].	67/female	Yes	Signet-ring cell adenocarcinoma; tubular adenocarcinoma (associated with gastrointestinal stromal tumor)	Not detected	No recurrence or metastasis for the following 8 years
Nugent SL, et al. [5].	64/female	Yes	Signet-ring cell adenocarcinoma	Detected	Not described
Kim JH, et al. [6].	53/male	Yes (double primary)	(with GCA) Tubular adenocarcinoma, (without GCA) Micropapillary carcinoma; moderately diff. tubular adenocarcinoma	Detected	No recurrence or metastasis for the following 18 months but relapsed
Enomoto Y, et al. (the present case)	80/male	Yes	Moderately diff. tubular adenocarcinoma; mucinous adenocarcinoma	Detected	No recurrence or metastasis for the following 6 months

## Conclusions

In conclusion, GCA should be recognized as a rare form of gastric malignancy, particularly detected with other types of adenocarcinomas. Further case series are needed to understand the clinical significance of this disease.

## References

[1] The WHO Classification of Tumours Editorial Board (2019). WHO Classification of Tumours, 5th edition. Digestive System Tumours.

[2] Roy P, Chetty R (2010). Goblet cell carcinoid tumors of the appendix: an overview. World J Gastrointest Oncol.

[3] Caruso RA, Heyman MF, Rigoli L, Inferrera C (1998). Composite early carcinoma (ordinary adenocarcinoma, carcinoid, microglandular-goblet cell carcinoid, neuroendocrine mucinous carcinoma) of the stomach. Histopathology.

[4] Fujiyoshi Y, Kuhara H, Eimoto T (2005). Composite glandular-endocrine cell carcinoma of the stomach. Report of two cases with goblet cell carcinoid component. Pathol Res Pract.

[5] Nugent SL, Cunningham SC, Alexiev BA, Bellavance E, Papadimitriou JC, Hanna N (2007). Composite signet-ring cell/neuroendocrine carcinoma of the stomach with a metastatic neuroendocrine carcinoma component: a better prognosis entity. Diagn Pathol.

[6] Kim JH, Eom DW, Park CS (2016). A concurrence of adenocarcinoma with micropapillary features and composite glandular-endocrine cell carcinoma in the stomach. J Gastric Cancer.

